# Direct Attachment with Erythrocytes Augments Extracellular Growth of Pathogenic Mycobacteria

**DOI:** 10.1128/spectrum.02454-21

**Published:** 2022-03-16

**Authors:** Yukiko Nishiuchi, Yoshitaka Tateishi, Hiroshi Hirano, Yuriko Ozeki, Takehiro Yamaguchi, Mari Miki, Seigo Kitada, Fumito Maruyama, Sohkichi Matsumoto

**Affiliations:** a Toneyama Institute for Tuberculosis Research, Osaka City University Graduate School of Medicine, Toyonaka, Japan; b Office of Industry-Academia-Government and Community Collaboration, Hiroshima University, Higashi-Hiroshima, Japan; c Department of Bacteriology, Niigata University Graduate School of Medicine, Niigata, Japan; d Department of Diagnostic Pathology, Tokyo Medical University Hachioji Medical Center, Tokyo, Japan; e Department of Pharmacology, Osaka City University Graduate School of Medicine, Osaka, Japan; f National Hospital Organization, Osaka Toneyama Medical Center, Toyonaka, Japan; g Laboratory of Tuberculosis, Institute of Tropical Disease, Universitas Airlangga, Surabaya, Indonesia; Quest Diagnostics

**Keywords:** *Mycobacterium avium* complex, tuberculosis, human erythrocytes, extracellular infection, complement receptor 1

## Abstract

Pathogenic intracellular mycobacteria, such as Mycobacterium tuberculosis and Mycobacterium avium, which cause lung diseases, can grow in macrophages. Extracellular mycobacteria have been reported in the lungs, blood, and sputum of patients, indicating the involvement of these pathogens in disease progression. Erythrocytes are involved in the symptoms associated with pulmonary mycobacterial diseases, such as bloody sputum and hemoptysis; however, little attention has been paid to the role of erythrocytes in mycobacterial diseases. Herein, we found that Mycobacterium avium subsp. *hominissuis* (MAH) and Mycobacterium intracellulare colocalized with erythrocytes at the sites of lung infection, inside capillaries and necrotic areas of granulomas, using histopathological examinations. Electron microscopy showed that MAH adhered and entered human erythrocytes when they were cocultured *in vitro*. MAH adhered to erythrocytes through complement receptor 1 and cell-surface sialo-glycoproteins. Importantly, MAH grew vigorously without causing any pronounced damage to erythrocytes. This erythrocyte-mediated enhancement of MAH growth occurred extracellularly depending on its direct attachment to erythrocytes. In contrast, MAH failed to multiply inside erythrocytes. Similarly, erythrocytes augmented the growth of other pathogenic mycobacteria, such as M. intracellulare and M. tuberculosis. THP-1 cell-derived human macrophages preferentially phagocytosed erythrocytes that were attached to mycobacteria (compared to bacteria alone), suggesting that erythrocyte-attached mycobacteria are an efficient infectious source for macrophages. Our findings provide new insights into the pathogenesis of mycobacterial diseases and offer an alternative and useful strategy for treating mycobacterial disease.

**IMPORTANCE** Pathogenic mycobacteria, such as Mycobacterium tuberculosis, Mycobacterium avium subsp. *hominissuis* (MAH), and Mycobacterium intracellulare, cause pulmonary infections as intracellular parasites of lung macrophages and epithelial cells. Here, using histopathological examinations we found that MAH and M. intracellulare colocalized with erythrocytes in lung infection sites. Subsequent studies demonstrated that direct interaction with erythrocytes enhances the extracellular proliferation of mycobacteria based on the following results: 1. MAH adhered and invaded human erythrocytes upon coculture *in vitro*; 2. MAH adhered to erythrocytes through complement receptor 1 and cell-surface sialo-glycoproteins; 3. MAH rapidly proliferated when directly attached to erythrocytes but not within them; 4. other mycobacteria, such as M. intracellulare and M. tuberculosis, also proliferated in the same way as MAH. The finding that pathogenic mycobacteria grow extracellularly in an erythrocyte-dependent manner is of considerable clinical importance for understanding disease progression and latent infection.

## INTRODUCTION

Mycobacterial diseases, namely, tuberculosis and nontuberculous mycobacteria (NTM) lung disease, pose threats to global public health (1–5). Mycobacterium tuberculosis was responsible for 1.4 million deaths in 2019 (1) and NTM is known to cause pulmonary infections in both immunocompromised and immunocompetent individuals, with a marked increase in incidence (2–5). Among them, Mycobacterium avium subsp. *hominissuis* (MAH) is one of the most common NTMs causing chronic lung diseases in humans. These pathogenic mycobacteria are believed to predominantly function as intracellular parasites in macrophages and epithelial cells. However, extracellular mycobacteria are often observed at the site of mycobacterial infections.

Numerous extracellular M. tuberculosis bacilli have been reported at the central necrotic area of mature granulomas, and a few have been reported at the rim of mature granulomas where foam cells and giant cells are located (6). In the granuloma region, macrophages are abundant and are known to polarize into M1 or M2 phenotypes, which play critical roles in killing pathogens and resolving inflammation, respectively. One or both of these phenotypes may be involved in the extracellular localization and proliferation of mycobacterial bacilli. In the caseous core of necrotic granulomas, M. tuberculosis bacilli are thought to replicate slowly or to exist in a nonreplicating state (7), suggesting the potential for latent infection. Moreover, it has been reported that extracellular M. tuberculosis can proliferate markedly in liquefied caseous granulomas and in open cavities (6, 7), representing active infection. As the liquefied regions of caseous granulomas and open cavities are known to be connected to the airways and blood vessels (8), bacilli can be released into the airways through coughing, and encounter erythrocytes in blood vessels. Consequently, mycobacterial cells in the blood can survive and proliferate extracellularly (9). Thus, extracellular bacilli may contribute significantly to the progression of disease. It is well known that extracellular M. avium or M. tuberculosis are present in the blood of patients with disseminated infections; hematogenous dissemination of M. tuberculosis and M. avium can occur at many sites, but the spleen, liver, and bone marrow are the most common sites of dissemination (10, 11). In addition, recent studies have demonstrated that extracellular mycobacteria have the potential to cross the blood-brain barrier (12). Taken together, many extracellular mycobacteria have been observed at infection sites, indicating their involvement in disease progression, and have been shown to be capable of extracellular proliferation; however, these mechanisms remain to be further elucidated.

When mycobacteria grow extracellularly, they are likely to encounter erythrocytes. Several reports have indicated the interaction between mycobacteria and erythrocytes, including: 1. bloody sputum and hemoptysis are one of the most common symptoms associated with pulmonary mycobacterial diseases; 2. anemia is a common complication of tuberculosis and disseminated M. avium infections (11, 13); 3. granuloma formation is associated with angiogenic processes, which serve as a source of immune cells, oxygen, nutrients for pathogens, and erythrocytes (14); and 4. necrotic areas of the granuloma contains both large bronchi and thrombosed blood vessels, serving as a likely sources of hemorrhage and an escape routes for the contents of the cavity into the environment (6, 14, 15).

Furthermore, the primary role of erythrocytes is transporting gases between the lungs and tissues. In addition, human erythrocytes play a role in defense mechanisms by eliminating invading pathogens from the circulation. Human erythrocytes capture pathogens using complement receptor 1 (CR1) and transfer them, along with CR1, to resident macrophages in the liver and spleen, while the remaining erythrocytes (those with reduced CR1) return to the circulation (16, 17). Although these findings suggest that mycobacteria interact with erythrocytes and influence virulence and infection defense, little attention has been paid to their roles in mycobacterial infections.

The purpose of this study was to clarify the mechanism by which erythrocytes interact with mycobacteria and their role in mycobacterial infection. We found that clinical mycobacterial strains colocalized with erythrocytes in lung infection sites, including inside capillaries and necrotic areas of granulomas. Our findings provide evidence that mycobacteria have the potential to adhere to erythrocytes for extracellular proliferation.

## RESULTS AND DISCUSSION

### Mycobacteria adhered to erythrocytes via complement receptor 1 (CR1) and sialo-glycoproteins.

**FIG 1:**
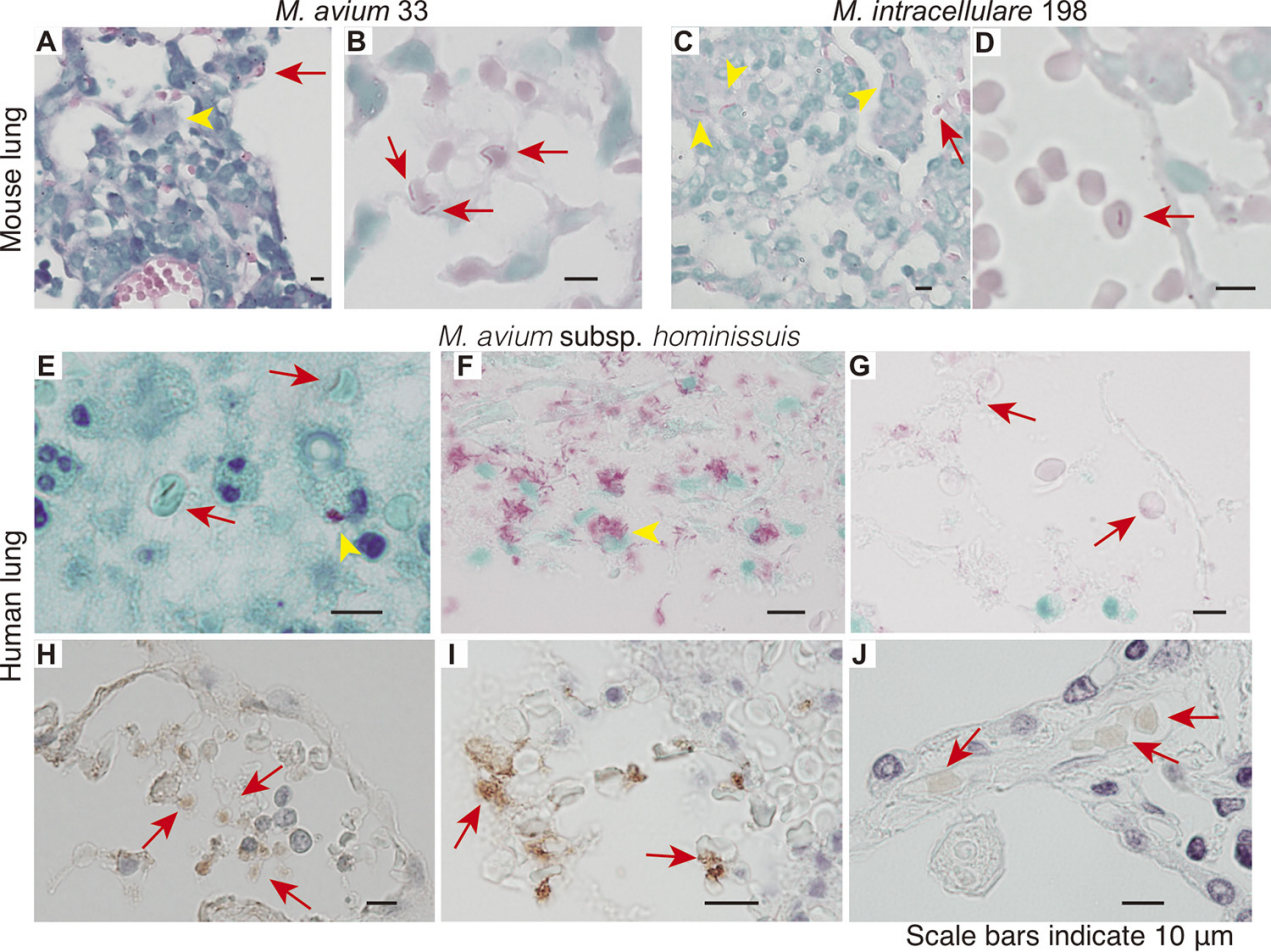
Histopathological examination of mouse and human lung tissues infected with M. avium or M. intracellulare. Lung tissues were obtained from mice infected with (A, B) M. avium 33 and (C, D) M. intracellulare 198 at 4 weeks postinfection, and (E–J) from the resected surgical specimen of a patient with pulmonary MAH infection. Paraffin-embedded lung sections were stained with (A–G) Ziehl–Neelsen stain, (H) an anti-antigen-85B antibody, (I) an anti-MDP-1 antibody, or (J) an anti-BCG antibody. Mycobacteria were observed to colocalize with erythrocytes (arrows) and macrophages (arrowheads). Original magnification: A, C, and F, 400×; B, D, E, and G–J, 1,000×.

We examined the histopathology of mouse lungs 4 weeks after infection with M. avium and M. intracellulare. Ziehl–Neelsen staining, which can identify acid-fast organisms, such as mycobacteria, showed that M. avium and M. intracellulare were present inside the macrophages (Fig. 1A and C, arrowhead); it also revealed the colocalization of these mycobacteria with erythrocytes in vasodilated alveolar capillaries, in which the erythrocytes were not deformed (Fig. 1A–D, arrow). We also observed that MAH colocalized with human erythrocytes in the necrotic granuloma regions of lung specimens surgically resected from a patient infected with MAH (Fig. 1E–G, arrow). In this region, we observed erythrocytes and numerous extracellular MAH (Fig. 1E–G). Specific mycobacterial antigens were detected on the erythrocytes using polyclonal antibodies against antigen-85B (Fig. 1H) and mycobacterial DNA-binding protein 1 (MDP-1) (Fig. 1I). An anti-Mycobacterium tuberculosis var. *bovis* BCG (BCG) polyclonal antibody-stained human erythrocytes in alveolar capillaries (Fig. 1J). These mycobacterial antibodies are specific for M. tuberculosis and are also cross-reactive for MAH (18, 19). Together, these data demonstrate that erythrocytes and mycobacterial pathogens do indeed interact *in vivo*.

**FIG 2:**
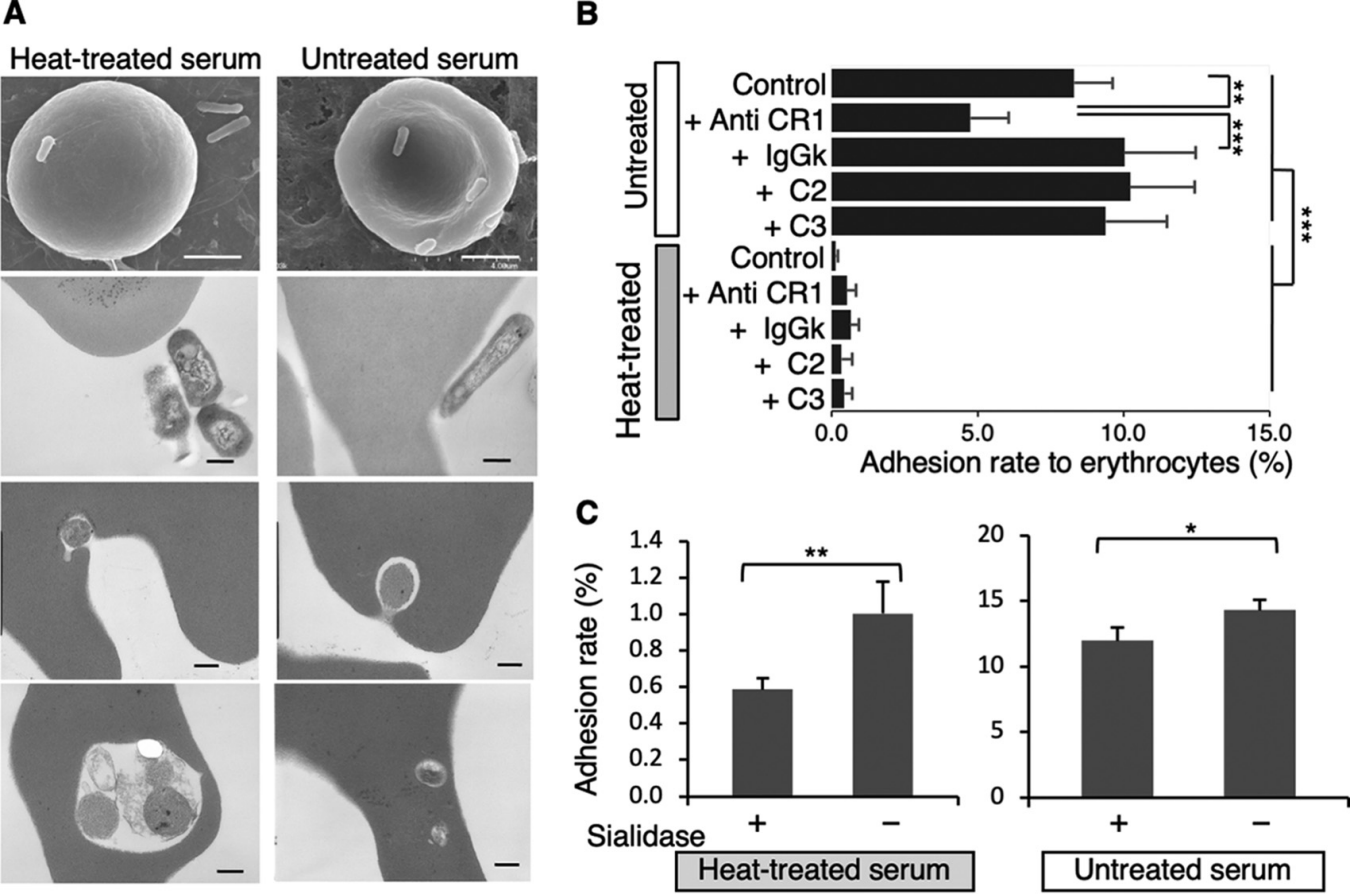
Attachment and invasion of MAH to human erythrocytes. (A) Images of MAH attachment/invasion into human erythrocytes using SEM and TEM analysis. The culture medium was supplemented with heat-treated (left) or untreated serum (right). White and black bars indicate 2 μm and 200 nm, respectively. (B) The adhesion rate of MAH to erythrocytes was determined in the presence of 10 μg/mL anti-human CR1 antibody, a control antibody (IgGk), or complement component 2 or 3. The adhesion rates were compared between culture media (containing untreated or heat-treated serum) using Kruskal–Wallis rank-sum tests (***, *P* = 6.46e − 14). The adhesion rates were also compared between the antibody- and complement-treated groups using one-way analysis of variance (ANOVA; *P* = 0.000012) followed by Dunnett’s multiple-comparisons test and generating adjusted *P* values via a single-step method (**, *P* = 0.0047 control versus + anti-CR1 antibody; ***, *P* < 0.001 control antibody [IgGk] versus + anti-CR1 antibody). (C) Removal of cell-surface sialic acids from erythrocytes by sialidase reduced the adhesion rate. **, *P* = 0.0052, *, *P* = 0.013 using two-tailed Student's *t* test. (B, C) The error bars indicate standard deviations (SDs; *n *= 3–4).

Next, we verified the interaction of MAH with human erythrocytes using scanning electron microscopy (SEM) and transmission electron microscopy (TEM). These analyses revealed that MAH attached to—and invaded—erythrocytes (Fig. 2A) after coculturing human erythrocytes with MAH in Roswell Park Memorial Institute (RPMI) 1640 medium supplemented with 10% human serum, either untreated or heat-treated. MAH exhibited a higher frequency of close contact with erythrocyte membranes in the culture medium containing untreated serum than in medium containing heat-treated serum (Fig. 2A). When the medium contained untreated serum, the adhesion rate was significantly higher than that when the medium contained heat-treated serum (Fig. 2B; *P* = 6.46e − 14). These observations could be attributable to MAH adhesion via CR1 expressed on human erythrocytes (16, 17), since complement proteins are inactivated in heat-treated serum. Inhibition experiments using anti-CR1 antibodies showed that the anti-CR1 antibody significantly, albeit incompletely, inhibited MAH adhesion (Fig. 2B; *P* = 0.0047; control versus anti-CR1 antibody; *P* < 0.001 control antibody versus anti-CR1 antibody).

Incomplete inhibition was suggestive of the presence of adhesion factors other than CR1. We hypothesized that sialo-glycoproteins could be involved in the interaction, because erythrocyte membranes contain abundant sialo-glycoproteins (also known as glycophorins) (20, 21). Actually, influenza virus, Plasmodium falciparum merozoites, and *Mycoplasma* target sialo-glycoproteins to attach to—and invade—host cells (22–25). To assess whether sialo-glycoproteins function as adhesion factors, we enzymatically removed sialic acids from sialo-glycoproteins, in accordance with a reported method (25), which significantly reduced the adhesion rate of MAH in both media (Fig. 2C and *P* = 0.0052 and *P* = 0.013; determined in the medium containing heat-treated and untreated serum, respectively). These results indicate that both CR1 and sialo-glycoproteins were involved in mediating the adhesion of MAH to erythrocytes.

Blocking CR1 and removing sialo-glycoproteins could not completely abrogate MAH-erythrocyte interaction, suggesting that these molecules cannot fully explain the adhesion mechanisms. With respect to the molecules of MAH responsible for the adhesion, we tested well-known mycobacterial adhesins, such as DnaK, Cpn60.2, extracellular DNA, trehalose dimycolate, glycopeptidolipid, heparin-binding hemagglutinin, and MDP-1. However, no contribution of any of these molecules to MAH adhesion was observed (S1 text and Fig. S1), suggesting that a mycobacterial adhesin—adhesins other than those tested above—may be necessary for MAH adhesion to erythrocytes.

### Erythrocytes promote extracellular, but not intracellular mycobacterial growth.

**FIG 3:**
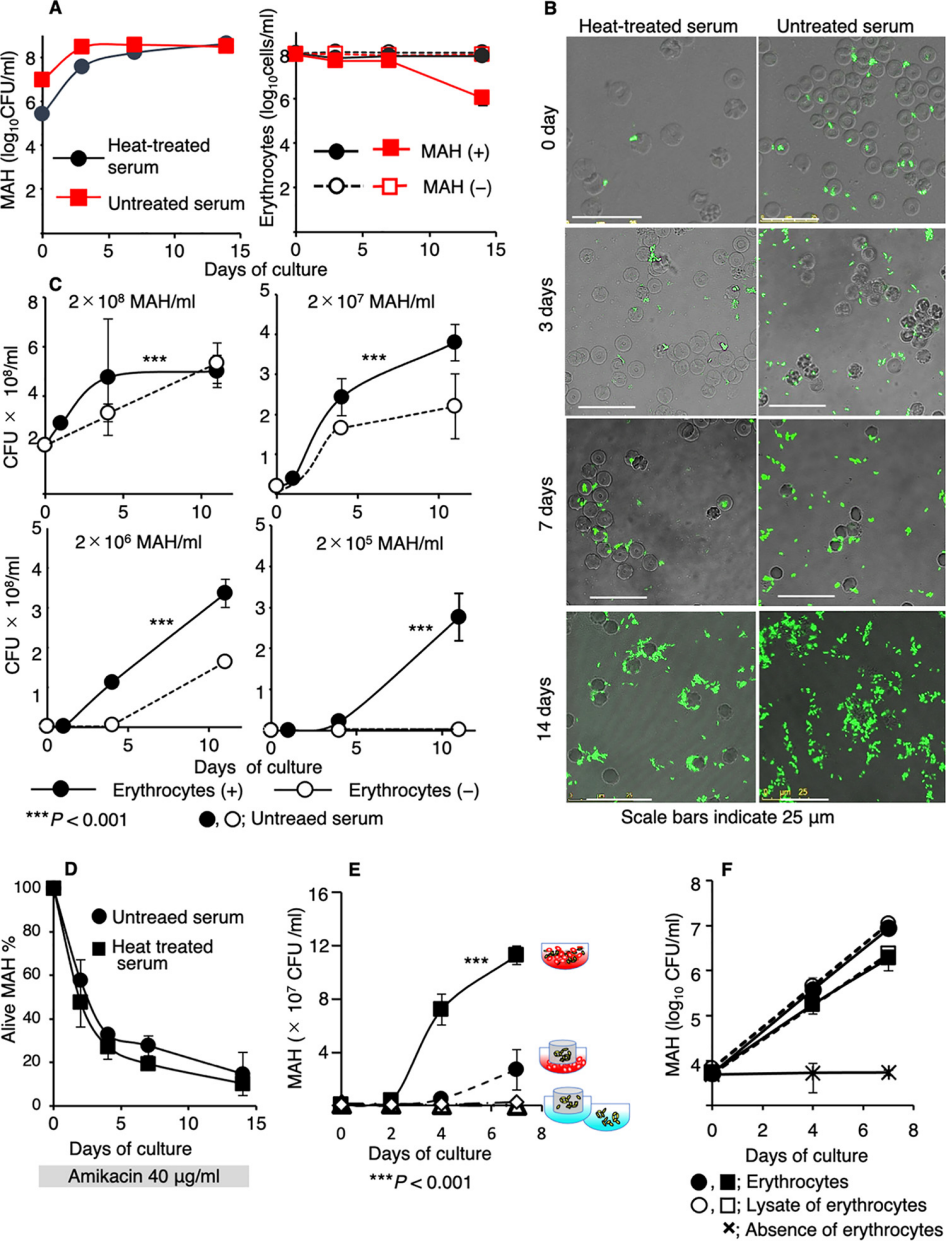
Extracellular growth of MAH. (A) Multiplication of MAH cells attached to erythrocytes. MAH (left panel) and erythrocyte cell counts (right panel). Erythrocytes were cultured in the presence or absence of MAH. (B) Microscopic images of the MAH cells and erythrocytes described in panel A. (C) Effect of the MAH inoculation size (2 × 10^5^–2 × 10^8^ cells/mL) on its growth in the presence or absence of erythrocytes (1 × 10^8^ cells/mL). (D) Reduction of the number of live MAH cells following amikacin treatment. (E) Promotion of MAH extracellular growth via the direct attachment to erythrocytes. The culture conditions are indicated. MAH cells coexisting with erythrocytes (squares); MAH cells separated from erythrocytes via cell culture inserts (CIs; circles); no erythrocytes, with (triangles) or without CIs (diamonds). (F) Growth of MAH coexisting with whole or hemolyzed erythrocytes. MAH cells (1 × 10^5^/mL) were inoculated into medium containing whole erythrocytes, erythrocyte lysate, or no erythrocytes. We included 1 × 10^8^ (circles) or 1 × 10^6^ (squares) erythrocyte cells or hemolyzed erythrocyte cell equivalents/mL. (C, E, F) All assays were conducted in culture medium with untreated serum. (A, C–F) The error bars indicate SDs (*n *= 3–4); ***, *P* < 0.001, as determined using Friedman’s rank-sum test.

To assess the viability of mycobacteria adhering to human erythrocytes, MAH growth assays were performed using a colony forming unit (CFU)-counting method. Green fluorescent protein (GFP)-expressing MAH was incubated with human erythrocytes for 1 h at a 1:1 ratio. After removing unbound MAH, erythrocytes with attached bacteria were cultivated for 14 days. MAH growth exponentially increased up to 3 days after coculture in both untreated and heat-treated serum-containing media, after which the growth transitioned to the stationary phase (Fig. 3A, left panel). Microscopic observations also showed that the quantity of MAH increased considerably over time in both settings (Fig. 3B). The proliferation rates of MAH obtained in both media were comparable (Fig. 3A, left panel, first 3 days), even though the respective adhesion rates to erythrocytes were significantly different, suggesting that MAH proliferation might proceed in a CR1-independent manner.

To evaluate the effect of the MAH inoculum size on its multiplication, MAH was cultured at different concentrations (2 × 10^5^–2 × 10^8^ cells/mL), with or without erythrocytes in medium containing untreated serum, and the growth kinetics were investigated by performing CFU assays (Fig. 3C). Significant MAH growth was observed in the presence of erythrocytes at all inoculated concentrations (*P* < 0.001) compared with that in the absence of erythrocytes. At a low initial inoculum concentration (2 × 10^5^ cells/mL), the MAH cells did not grow in the absence of erythrocytes. MAH multiplied in an erythrocyte-dependent manner with a generation time of 11 h. This generation time is comparable to that of MAH in an optimized culture medium (10–12 h), and faster than that of MAH multiplication inside human-derived macrophages (20–108 h) (26). These results demonstrate that erythrocytes promoted the vigorous growth of MAH.

The number of erythrocytes was unchanged up to 7 days after culturing in both media, regardless of the presence or absence of MAH (Fig. 3A, right panel). Beyond a week after cultivation, the number of erythrocytes gradually decreased when untreated serum was used, probably due to the occurrence of hemolysis (Fig. 3A right, 3B). Thus, no hemolysis was observed during MAH growth; however, hemolysis occurred after the growth peak. During these cultivation periods, no hemagglutination was observed under microscopic observation (Fig. 3B) and hemagglutination assays (Fig. S2A). Compared with erythrocytes cultured alone, erythrocytes cocultured with MAH showed no significant reduction in the resistance to osmotic fragility after 1 h or 3 days of culture (Fig. S2B–G). Thus, the adhesion of MAH to human erythrocytes caused only minimal damage to erythrocyte membranes and caused vigorous MAH proliferation. As these observations fulfill all the requirements for infection—the invasion and multiplication of microorganisms that are not normally present within the body—we concluded that MAH cells are capable of infecting erythrocytes.

Next, we investigated the potential of MAH to grow inside erythrocytes. When erythrocytes cocultured with MAH were treated with amikacin, a membrane-impermeable bactericidal agent, the total number of MAH cells decreased over time (Fig. 3D). TEM observations showed that the envelopes of MAH cells engulfed by erythrocytes were damaged, even without amikacin treatment (Fig. 2A). These results clearly indicate that MAH cannot survive inside erythrocytes. Additionally, we assessed whether direct interaction with erythrocytes is necessary for erythrocyte-dependent extracellular proliferation of MAH (Fig. 3E). Using cell culture inserts (CIs), we examined MAH proliferation by separating MAHs from erythrocytes in the culture medium (with CIs), by direct interaction (without CIs), or in the absence of erythrocytes (with/without CIs). The results revealed active MAH cell proliferation when the cells directly interacted with erythrocytes (Fig. 3E and *P* = 0.00012). Even when the erythrocytes were replaced with their lysates in the culture medium, the MAH cells grew well with comparable growth rates (Fig. 3F). These results indicate that MAH growth on the outside of the erythrocytes was highly dependent on its direct interaction with erythrocytes and/or erythrocyte components. Regarding bacterial growth, iron is known to be an essential nutrient, and some bacteria, including M. tuberculosis and *Mycolicibacterium smegmatis*, can utilize heme and/or hemoglobin as iron sources (27, 28). It is possible that MAH cells attached to erythrocytes directly capture heme and/or hemoglobin as iron sources.

**FIG 4:**
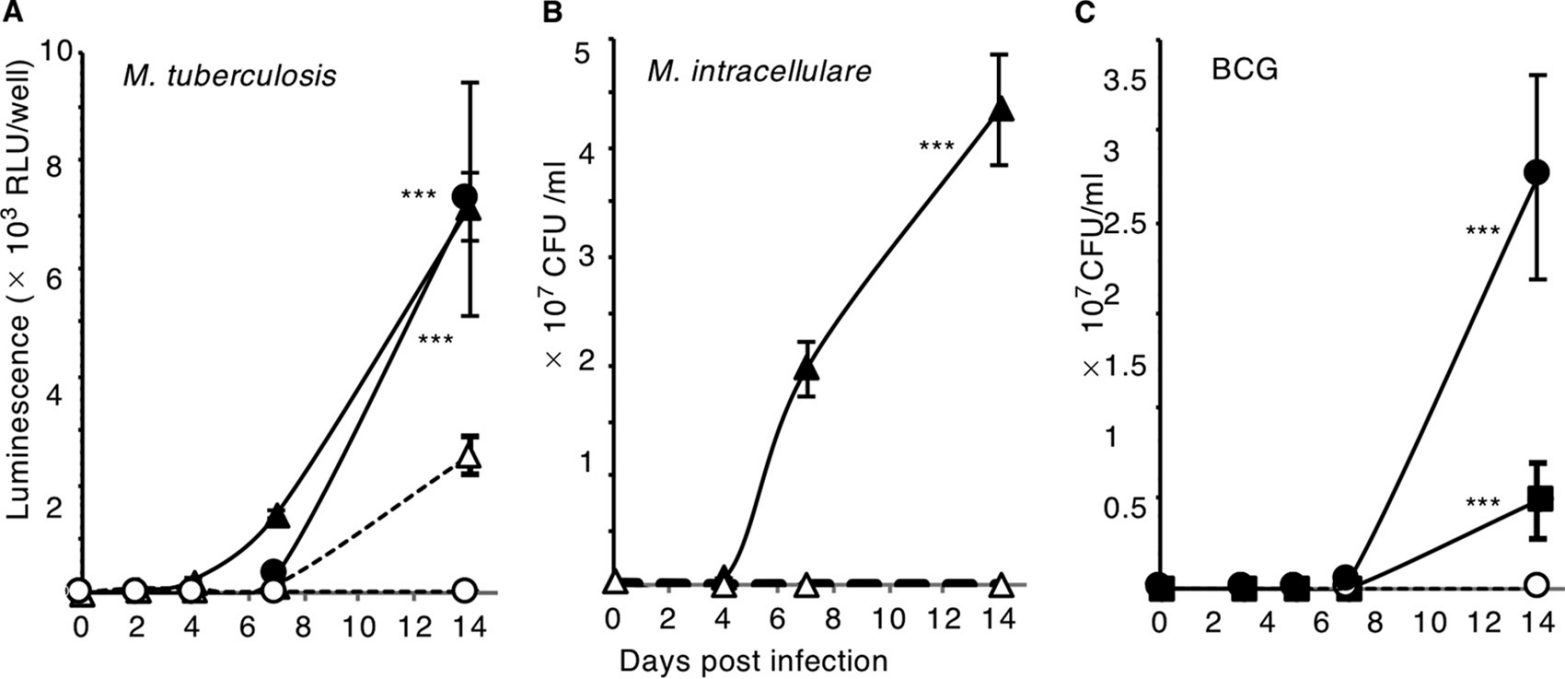
Erythrocytes promoted the growth of other mycobacteria. (A–C) Pathogens were inoculated into the media in the presence (solid markers) or absence (open markers) of erythrocytes. ***, *P* < 0.001, as determined by two-way repeated measures ANOVA. (A) M. tuberculosis H37Rv cells expressing luciferase were challenged at 2 × 10^6^ CFU/mL (triangles) or 2 × 10^5^ CFU/mL (circles), after which the relative light units (RLUs) were measured. (B) M. intracellulare 198 challenged at 1 × 10^6^ CFU/mL (triangles). (C) BCG challenged at 3 × 10^5^ CFU/mL (circles) or 3 × 10^4^ CFU/mL (squares). Assays were conducted in culture medium with untreated serum.

Erythrocytes promoted the growth of all tested mycobacterial species: M. tuberculosis, M. intracellulare, and BCG (Fig. 4A–C). As with MAH, we confirmed that (i) BCG could directly attach to and invade erythrocytes (Fig. S3A–D), (ii) M. intracellulare could not survive inside erythrocytes (Fig. S3E), (iii) direct attachment of M. tuberculosis to erythrocytes was essential for its own multiplication (Fig. S3F, G), and (iv) no decrease in erythrocyte osmotic fragility occurred after the addition of M. intracellulare (Fig. S3H–M). These results demonstrate that the vigorous extracellular growth induced by erythrocytes is a common property of pathogenic mycobacterial species.

### Macrophages preferentially engulfed erythrocytes with MAH attached over MAH alone.

**FIG 5:**
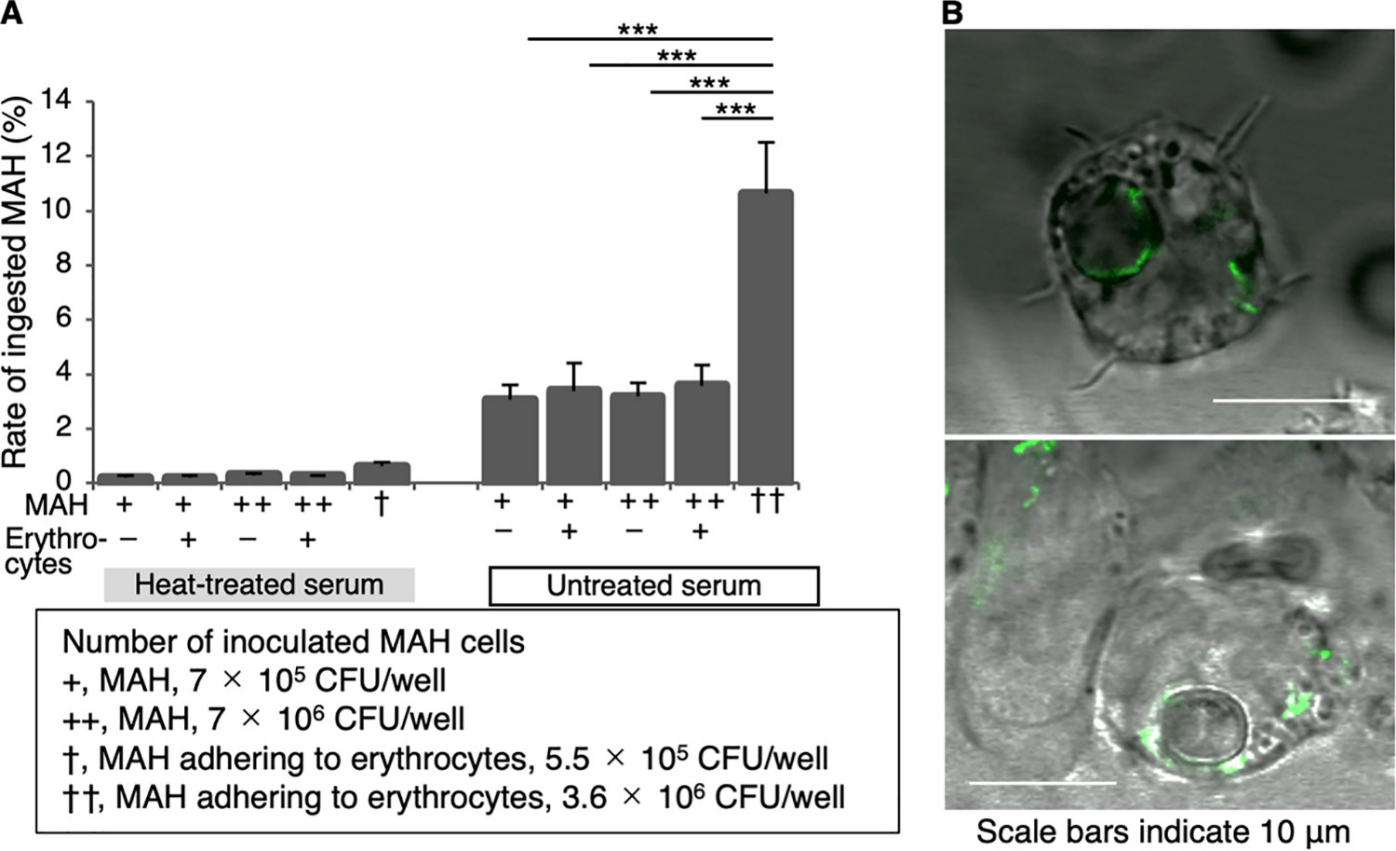
Macrophages preferentially engulfed erythrocytes with attached MAH cells over MAH cells alone. (A) Promotion of macrophage phagocytosis of MAH cells adhering to erythrocytes. The ratio of the number of MAH cells engulfed by THP-1 monocyte-derived macrophages to the number of inoculated MAH cells is presented. MAH cells were inoculated under the following three situations: MAH adhering to erythrocytes, MAH alone, and MAH plus erythrocytes. ***, *P* < 0.001, as determined using Dunnett's multiple-comparisons test; *P* values were adjusted using a single-step method. (B) Confocal microscopy images of macrophages that engulfed MAH cells (green) adhering to erythrocytes. All error bars indicate SDs (*n *= 3–4).

Macrophages are target cells for infection by mycobacteria (29), although they are intrinsically capable of phagocytosing microbes and killing virus-infected cells, clearing dead and senescent cells, and repairing damaged tissues (29–32). Further, we assessed the effect of MAH-attached erythrocytes on the phagocytic capability of macrophages compared to MAH cells alone. The extent of phagocytosis of MAH cells was expressed as the ratio of the number of MAH cells engulfed by THP-1 monocyte-derived macrophages to the number of inoculated MAH cells. Macrophages engulfed more MAHs attached to erythrocytes than did MAH cells alone, as determined by examining the extent of phagocytosis (Fig. 5A and *P* < 0.001) and confocal microscopy analysis (Fig. 5B). Melhrn et al. demonstrated that pathogen binding to CR1 on human erythrocytes promotes ATP release and consequently stimulates the phagocytosis of immune-adherent immune complexes (33). Our results may also be influenced by these effects. These findings suggest that erythrocyte-attached mycobacteria could be an efficient infectious source for macrophages.

### Extracellular infection and pathogenesis.

Our findings demonstrate that pathogenic mycobacteria adhered to human erythrocytes through CR1 and sialo-glycoproteins on erythrocytes and rapidly proliferated. We clarified that this proliferation required direct interaction with human erythrocytes or the presence of components of erythrocytes. During mycobacterial proliferation, there was no significant damage to the cell membrane of erythrocytes. These results revealed the involvement of erythrocytes in the extracellular proliferation of mycobacteria. In other words, pathogenic mycobacteria can infect human erythrocytes and promote extracellular proliferation. Unfortunately, our study did not reveal adhesins on the surface of mycobacterial cells, although they may be an important factor explaining the active growth of mycobacteria, and thus, should be clarified in future studies.

In the present study, erythrocytes with/without attached mycobacteria and numerous extracellular MAH cells were observed in the necrotic granuloma region of a pulmonary MAH disease patient. Previous studies have also reported extracellular M. tuberculosis cells in such regions (6). These extracellular mycobacteria are thought to result from the necrotic breakdown of macrophages. In addition, polarized M1-like macrophages are abundantly present in the necrotic granuloma region, whereas M2-like macrophages are abundant at the rim of mature granulomas (34). Furthermore, it has been reported that mycobacteria grow vigorously in an extracellular milieu created by the absence of macrophages or through macrophage necrosis (6). Our findings, along with those of previous studies, suggest that macrophage polarization may affect the growth of extracellular mycobacteria as well as mycobacteria within macrophages. Consequently, granulomas may serve as a niche for mycobacteria to proliferate extracellularly by interacting with erythrocytes. Mycobacterial infection of erythrocytes may also be involved in anemia. Most patients with disseminated MAH disease experience severe anemia (10), whereas tuberculosis-associated anemia is usually mild and resolves with antituberculosis treatment (13, 35). One possibility is that infected erythrocytes may promote their phagocytosis by macrophages, as shown by our results, which may lead to anemia.

### Possible role for the attachment of mycobacteria to erythrocytes in pathogenesis.

We showed that erythrocytes are present in the capillaries and necrotic granulomas of lungs from mice and human (Fig. 1). *In vitro* studies demonstrated that erythrocytes promoted the extracellular growth of mycobacteria (Fig. 3 and 4). Erythrocyte-dependent extracellular proliferation may contribute to the abundance of mycobacterial cells at the center of necrotic granulomas.

The mycobacterial attachment to the erythrocytes suggests a role in the hematogenous dissemination of mycobacterial pathogens through releasing into blood vessels from liquefied caseous granulomas and open cavities. Most microbes captured by erythrocytes through CR1 are decayed by resident macrophages in the liver and spleen. Unlike other microbes, mycobacteria can survive and proliferate in this environment; thus, hematogenous dissemination may lead to disease exacerbation. This hypothesis is supported by the fact that hematogenous dissemination of mycobacteria is more frequently observed in the liver and spleen compared with other organs (9, 10).

We showed that the erythrocytes capture of mycobacteria is partially dependent on CR1 (Fig. 2). It was also considered that adhesion factors other than CR1 (Fig. 2) may prevent migration to the liver and spleen, thus promoting the circulation of mycobacterial pathogens throughout the body. Such circulating erythrocyte-attached mycobacteria will ultimately be engulfed by tissue-resident macrophages, as we observed that THP1 cell–derived macrophages preferentially phagocytosed mycobacteria-erythrocyte complex to mycobacteria alone (Fig. 5). All tested mycobacterial strains in this study, such as M. tuberculosis, BCG, MAH, and M. intracellulare adhered to erythrocytes and experienced high levels of proliferation in the presence of erythrocytes, suggesting that adhesion to erythrocytes and subsequent augmented proliferation are common for mycobacteria. Understanding the adhesion of mycobacteria to erythrocytes will help to elucidate the mechanisms underlying the systemic dissemination of mycobacteria and their role in disease progression.

### Two facets of the interaction between erythrocytes and mycobacteria.

Erythrocytes transport gases and eliminate pathogens from the host circulation (16, 17, 32, 33). Thus, it can be said that the role of erythrocytes in mycobacterial infection has two facets: erythrocytes play a defensive role against infection and could also be the target cells of mycobacterial infection. It is likely that the balance between these two facets is an important determinant of the outcome of mycobacterial infection. If the defense system optimally functions, mycobacterial diseases can be controlled or the incubation period can be prolonged. However, if the mycobacterial attack is overwhelming, mycobacterial diseases may develop and sometimes disseminated throughout the body. Our findings provide a platform to address the following unsolved issues of mycobacterial infections in future studies: (i) The incubation period of tuberculosis is long, sometimes decades, but the period has not been well characterized (36). 2) The mechanisms underlying the dissemination of MAC infection (10) and tuberculosis, which is known as miliary tuberculosis, are not well understood (9). 3) The mechanism by which anemia becomes associated with mycobacterial infection (10, 13, 35) is unclarified.

### Conclusion.

Pathogenic mycobacteria infected human erythrocytes and promoted their own extracellular multiplication. Our findings provide insights into new aspects of mycobacterial disease pathogenesis and immunity against mycobacterial infection, which offer alternative strategies for the development of mycobacterial disease therapies.

## MATERIALS AND METHODS

### Histopathology.

We used paraffin-embedded lung tissues prepared from a group of five mice infected with M. avium 33 or M. intracellulare 198 (37), and from surgically resected human lung specimens. The samples were obtained from a patient with MAH infection, who had undergone surgical resection in the Osaka Toneyama Medical Center. This experiment was approved by the Osaka Toneyama Medical Center Institutional Ethical Review Board for Human Subject Experimentation and complied with international guidelines for studies involving human subjects; all recruited individuals provided written informed consent. Sections were stained with the Ziehl–Neelsen stain, and immunohistochemical staining was performed using a rabbit polyclonal antibody against M. tuberculosis antigen-85B, a rabbit polyclonal antibody against M. tuberculosis MDP-1 (38), or a polyclonal anti-BCG antibody. The antigens 85B and MDP-1 are mycobacterium-specific proteins and show a high degree of homology between M. tuberculosis H37Rv and MAH 104 (85% and 97% of the N-terminal 103 amino acids, respectively). These antibodies show cross-reactivity with MAH (19). Immunohistochemistry was performed as previously described (38). Immunohistochemical staining with the anti-BCG antibody was outsourced to Kyodo Byori, Inc. (Kobe, Japan).

### Mycobacterial strains, antibodies, and culture conditions.

We used the following mycobacterial strains: MAH 104 strain expressing GFP (39); a rough variant of MAH 104 (MAH 104R), which was naturally derived from MAH 104 and does not produce glycopeptidolipid (39); M. intracellulare 198 (36); BCG Tokyo; and M. tuberculosis H37Rv expressing luciferase (40). Mycobacterial strains were grown at 37°C in Middlebrook 7H9 broth (BD Biosciences [BD], Franklin Lakes, NJ) supplemented with 10% Middlebrook albumin-dextrose-catalase enrichment (BD) and 0.2% (vol/vol) glycerol or 0.05% (vol/vol) Tween 80. Viability was determined by counting CFU on Middlebrook 7H11agar plates supplemented with 0.5% (vol/vol) glycerol and 10% Middlebrook oleic acid-albumin-dextrose-catalase enrichment (BD), with 10-fold serial dilutions. Seven days after cultivation, the colonies were counted using a stereo microscope. Luciferase activities in bacterial suspensions (50 μl) were measured for 15 s using a LUMAT LB 9507 luminometer (Berthold Bad Wildbad, Germany). Mycobacteria were grown for 7 to 14 days and resuspended in saline. Then, they were adjusted to an OD_660_ of 1 (corresponding to 1 × 10^9^ CFU/mL) in saline prior to coculturing with erythrocytes. The following antibodies were used at a concentration of 10 μg/mL: a purified mouse anti-human CD35 antibody (anti-CR1, 558768, BD Bioscience) and a mouse IgG1 κ isotype control antibody (554121, BD Bioscience).

### Human erythrocytes and culture conditions.

Blood samples from donors were kindly provided by the Japanese Red Cross Kinki Block Blood Center of the Japanese Red Cross Society. This research was approved by the Institutional Ethical Review Board of Osaka City University. Blood donors agreed to a comprehensive analysis with written informed consent. Serum was prepared from plasma by removing clots after adding CaCl_2_ (final concentration: 10 mmol/l) and incubating the plasma for 30 min at 37°C. A portion of the serum was treated by heating at 56°C for 30 min. Untreated and heat-treated sera were stored at −20°C until use. Erythrocytes were washed three times with sterile saline and centrifuged at 800 × *g* for 8 min at 4°C. Next, the erythrocytes were plated at a concentration of 1 × 10^8^ cells/mL suspension in RPMI 1640 medium supplemented with 25 mM HEPES and 10% heat-treated or untreated human serum on petri dishes or 24-well tissue culture plates.

### Coculturing mycobacteria with human erythrocytes.

Erythrocytes were cultured overnight in a 5% CO_2_ incubator at 37°C prior to coculture. Human erythrocytes (1 × 10^8^ cells/mL) were cocultured with different concentrations of mycobacteria (2 × 10^5^–2 × 10^8^ cells/mL) for 1 h to 2 weeks. Unbound bacilli were removed from the erythrocytes by washing them with saline three or four times, as needed. The viabilities of mycobacteria and erythrocytes were assessed by CFU counting and by a hemocytometer. Cocultured samples were examined using a TCS-SP5 confocal laser-scanning microscope (Leica, Wetzlar, Germany). To kill extracellular bacilli, mycobacteria cocultured with erythrocytes were treated with 40 μg/mL amikacin throughout the culture period. The amikacin concentration and exposure time were determined by determining the minimum-inhibitory concentration and minimum-bactericidal concentration values of amikacin against MAH (Table S1, Fig. S4). To separate mycobacteria from erythrocytes, we used a CI with 0.4 μm pore size (BD Biosciences) in a 24-well plate. Following overnight preculture of erythrocytes (1 × 10^8^ cells/mL), mycobacteria were inoculated at a 1:100 ratio into the CI (to separate the mycobacteria from the erythrocytes), into wells without a CI (coexisting mycobacteria with erythrocytes), or in the absence of erythrocytes. Then, the cells were cultured for 7 to 11 days, and the number of mycobacterial cells was counted. To prepare medium containing components of hemolyzed erythrocytes, we added an equal volume of sterilized water to a concentrated erythrocyte suspension (2 × 10^9^ cells/mL), which was mixed well to permit complete hemolysis. The hemolyzed solution was diluted in culture medium to achieve the desired concentration (1 × 10^6^ or 1 × 10^8^ cell equivalents/mL). All experiments were performed with three or four biological replicates. The data shown are representative of at least two independent experiments.

### Rate of MAH adhesion to erythrocytes.

The adhesion rate was determined by dividing the number of mycobacteria bound to erythrocytes by the total number of mycobacteria inoculated in each well. We cocultured human erythrocytes with MAH organisms in medium supplemented with untreated or heat-treated serum for 1 h at 37°C, followed by washing erythrocytes with saline and centrifugation at 800 × *g* for 8 min at 4°C, three or four times. After washing, the erythrocytes were hemolyzed, and mycobacterial cell counts were assayed by determining the CFU. Erythrocytes were preincubated with 10 μg/mL of anti-human CR1 antibody or control antibody (IgGk) and complement components 3 or 2 for 30 min prior to the infection with MAH. For sialidase treatment, erythrocytes were incubated with 150 mU/mL sialidase (11080725001, Roche) at 37°C overnight, followed by washing the erythrocytes.

### SEM and TEM experiment.

For SEM experiments, erythrocytes were cocultured with mycobacteria for 1 h at a 1:1 ratio in RPMI 1640 culture medium supplemented with 10% untreated or heat-treated human serum. After removing unbound mycobacteria by washing, the erythrocytes were placed on coverslips (Thermanox Plastic coverslips, Thermo Fisher Scientific Inc., Waltham, MA, USA) and prefixed for 10 min in a solution of 2.5% (vol/vol) glutaraldehyde in 0.1 mol/l cacodylate buffer (CB, pH 7.4) and then rinsed three times in CB. Next, fixation and subsequent sample preparation were performed as previously described (39). The samples were observed using a S4700 scanning electron microscope (Hitachi Ltd., Tokyo, Japan).

For TEM preparation, erythrocytes cocultured with mycobacteria were prepared as described above and fixed for 1 h with 2.5% glutaraldehyde in CB. Erythrocytes were pelleted and embedded in agar. The agar blocks were fixed again with 2.5% glutaraldehyde for 1 h followed by three rinses with CB. Then, the blocks were resuspended in 1% (vol/vol) osmium tetroxide in CB for 1 h, rinsed twice with CB, and dehydrated using a graded-ethanol series (50, 70, 90, 99, and 100%) and propylene oxide (twice for 15 min). Next, the blocks were embedded using the Spurr Resin kit (Polysciences, Warrington, PA, USA), cut into thin sections, and stained with 5% uranyl acetate, followed by Reynold’s lead citrate. Stained sections were viewed using a JEM-1200EXII transmission electron microscope (JEOL, Tokyo, Japan).

### Phagocytosis assay with human THP-1 monocyte-derived macrophages.

We used the human monocytic THP-1 cell line, which was purchased from the Riken BioResource Research Center (Tsukuba, Japan). The cells were cultured in RPMI 1640 medium containing 10% heat-treated human serum, 100 units/mL of penicillin, and 100 μg/mL of streptomycin, and subcultured every 3 to 4 days. At 48 h prior to infection, THP-1 cells (5 × 10^5^ cells in 500 μl) were cultured in 24-well plates, and then differentiated in the presence of 100 nM phorbol 12-myristate 13-acetate (Sigma-Aldrich, St. Louis, MO, USA). Differentiated monolayered cells were washed three times with Dulbecco’s modified Eagle’s medium (DMEM) supplemented with 10% untreated or heat-treated human serum. THP-1 monocyte-derived macrophages were cocultured with MAH and erythrocytes. Coculture was performed in the following three ways: inoculated MAH cells were free in the presence or absence of erythrocytes or were adhered to erythrocytes. MAH cells adhering to erythrocytes were prepared by coculturing erythrocytes with MAH for 24 h, washing the erythrocytes to remove the unbound MAH, resuspending the erythrocytes in culture medium, and adjusting the erythrocytes to a concentration of 10^8^ cells/mL. The numbers of attached and free MAH cells in the culture media were counted by performing CFU assays. After incubation for 3 h to allow macrophages to phagocytose MAH or MAH to adhere to erythrocytes, the number of viable MAH cells engulfed by macrophages was determined by performing CFU assays. To determine the number of engulfed MAHs, the macrophages were washed four times with DMEM to remove the nonphagocytosed MAH cells and erythrocytes, after which they were lysed with 1% Triton X-100 in phosphate-buffered saline. The ratio of engulfed MAH was calculated by dividing the number of engulfed MAH cells by the number of inoculated cells.

### Statistical analysis.

Statistical analyses were performed using R software version 3.5.0 (2018-04-23) (41). The Bartlett test was used to verify the assumption that variances were equal across different groups or samples. When equal variance across samples was found, one-way or two-way repeated ANOVA was performed for repeated measures followed by Dunnett’s multiple-comparisons posttest. When unbalanced variance across samples was detected, the Kruskal–Wallis rank-sum test or Friedman’s test was used for repeated measures, followed by the pairwise Wilcoxon rank-sum test to calculate pairwise comparisons between groups, with corrections for multiple testing. To compare two independent groups with equal variances, a two-tailed Student's *t* test was used. Results with *P* < 0.05 were considered significant.
