# Supplementary material for: Direct Attachment with Erythrocytes Augments Extracellular Growth of Pathogenic Mycobacteria
Source: Microbiol Spectr. 2022 Mar 16;10(2):e02454-21. doi: 10.1128/spectrum.02454-21 (PMC9045221; doi:10.1128/spectrum.02454-21)
Supplement: SUPPLEMENTAL FILE 1 — Supplemental material. Download SPECTRUM02454-21_Supp_1_seq7.pdf, PDF file, 1.0 MB [file spectrum02454-21_supp_1_seq7.pdf]

Supplemental file

Direct attachment with erythrocytes augments extracellular growth of pathogenic mycobacteria

Yukiko Nishiuchi, Yoshitaka Tateishi, Hiroshi Hirano, Yuriko Ozeki, Takehiro Yamaguchi, Mari Miki, Seigo Kitada, Fumito Maruyama, Sohkichi Matsumoto

S1 text.

An undefined MAH adhesin molecule underlies adhesion to erythrocytes

We aimed to identify the molecules on the surface of MAH responsible for adhesion to erythrocytes. We examined the various adhesin candidates of MAH, such as DnaK, Cpn60.2, extracellular DNA, TDM, GPL, HBHA, and MDP1, that had been previously reported (1–8). However, none of these apparently mediated MAH adhesion to erythrocytes. Recent studies show that *M. tuberculosis* chaperon proteins, i.e., Cpn60.2 (Hsp65 GroEL2) and DnaK (Hsp70), which are present on the bacterial cell surface, could bind sialo-glycoproteins (1,2). Genes in the MAH 104 genome encode both proteins, and exhibit a high homology with *M. tuberculosis* genes (94% and 95% identity, respectively) recorded on the National Center for Biotechnology Information database. We therefore examined the effect of anti-Cpn60.2 and anti-DnaK antibodies on the adhesion of mycobacteria to sialo-glycoproteins on human erythrocytes. However, pre-treatment with anti-DnaK or anti-Cpn60.2 antibody did not cause a reduction in the adhesion rate to erythrocytes (Figure S1A). Therefore, Cpn60.2 and DnaK are not likely to underlie the binding of mycobacterial adhesin to erythrocytes.

Next, we examined the adherence of GPL, which is a major surface component possessing the ability to adhere to mouse lung epithelial cells (3) and to inert surfaces (4). Both MAH 104 and MAH 104R, a GPL deficient mutant strain, exhibited similar adhesion rates (Figure S1B). SEM and TEM showed that MAH 104R also attached to—and invaded—erythrocytes, as was the case for the wild type (Figure S1C). These results suggested that GPL may not affect the attachment of MAH to

erythrocytes. Another adhesin candidate is an extracellular DNA, which is known known to be an important biofilm component responsible for initial attachment in the biofilm formation of *Listeria monocytogenes* (5). We treated the extracellular DNA of MAH with 100 µg/mL DNase I in three ways, i.e., non-treatment, DNase treatment prior to infection for 30 min, and DNase treatment prior to—and throughout—the infection period. However, DNase treatment did not alter the rate of MAH adhesion to erythrocytes, suggesting that the extracellular DNA has no functional role as an adhesin (Figure S1D).

In addition, trehalose dimycolate (TDM), which binds to the Mincle receptor on the macrophages to induce inflammatory responses (6), was also not considered likely to affect the attachment of MAH to erythrocytes, as no erythrocytes were found to bind to the TDM-coated plate (0.01–10 µg TDM/well) (Figure S1E). Other adhesin candidates are HBHA and MDP1, which are abundantly expressed on the mycobacterial cell wall and bind to glycosaminoglycans, such as heparin, heparan sulfate, hyaluronic acid, and chondroitin sulfates (7,8). The adhesion of these proteins to glycosaminoglycans is known to be inhibited upon the addition of heparin or mannose (7,8). However, in the present study, heparin and mannose did not affect the adhesion rate of these proteins to erythrocytes (Figure S1F). Additionally, MDP1 showed no effect on the attachment of MAH to erythrocytes (Figure S1G). Furthermore, neither protein could hemagglutinate (Figure S1H). Although all of the molecules examined in the present study, i.e., Cpn60.2, DnaK, GPL, extracellular DNA, TDM, HBHA, and MDP1, are either an adhesin or a virulent factor that interact with host cells, our results indicate that they are not likely to be responsible for attaching MAH to human erythrocytes. Therefore, it is likely that an unknown molecule(s) is involved in the adhesion of MAH to erythrocytes.

MATERIALS AND METHODS

Rate of MAH adhesion to erythrocytes. To determine the effects of the antibodies on the adhesion rate, erythrocytes were preincubated with the following antibodies at a concentration of 10 µg/ml: an anti-Cpn60.2 antibody (anti-Hsp65, ab69618, Abcam®), and an anti-DnaK antibody (ab73473, Abcam®). The effect of GPL was examined using a GPL-deficient mutant strain (MAH 104R). To confirm the effect of extracellular DNA, extracellular DNA from MAH cells was degraded by treatment with 100 µg/ml DNase I at 37 °C. Trehalose dimycolate (TDM) and MDP-1 were purified as described previously (9). Heparin-binding hemagglutinin was purified as described previously (10).

Hemagglutination assay. The hemagglutination activity of human erythrocytes with MAH attached was assayed in V-shaped microtiter plates (Corning®, Armonk, NY) for 5 h at approximately 20–25 °C, in RPMI medium supplemented with untreated serum.

Osmotic fragility. The osmotic fragility of human erythrocytes with MAH attached was determined by lysis in a hypotonic-buffered saline, based on a previously reported method (11). Erythrocytes (1 × 10^8 cells) were suspended in 1 ml of hypotonic-buffered saline solutions, ranging from 0.3 to 0.9% (w/v) NaCl or water (100% lysis control), and incubated at room temperature for 30 min followed by centrifugation at 800 × g for 2 min. Next, 100 µl supernatants obtained from the treated solutions were transferred, and the rate of hemolysis was measured using a plate reader (iMark™ Bio-Rad, Hercules, CA) at 405 nm. The results were normalized to the 100% lysis control and osmotic-fragility curves were plotted.

REFERENCES

1. Hickey TB, Thorson LM, Speert DP, Daffé M, Stokes RW. 2009. *Mycobacterium tuberculosis* Cpn60.2 and DnaK are located on the bacterial surface, where Cpn60.2 facilitates efficient bacterial association with macrophages. Infect Immun 7: 3389-3401. doi: 10.1128/IAI.00143-09.
2. Hickey TB, Ziltener HJ, Speert DP, Stokes RW. 2010. *Mycobacterium tuberculosis* employs Cpn60.2 as an adhesin that binds CD43 on the macrophage surface. Cell Microbiol 12: 1634-1647. doi: 10.1111/j.1462-5822.2010.01496.x.
3. Yamazaki Y, Danelishvili L, Wu M, Hidaka E, Katsuyama T, Stang B, Petrosky M, Bildfell R, Bermudez LE. 2006. The ability to form biofilm influences *Mycobacterium avium* invasion and translocation of bronchial epithelial cells. Cell Microbiol 8, 806-814. doi: 10.1111/j.1462-5822.2005.00667.x.
4. Freeman R, Geier H, Weigel KM, Do J, Ford TE, Cangelosi GA. 2006. Roles for cell wall glycopeptidolipid in surface adherence and planktonic dispersal of *Mycobacterium avium*. Appl Environ Microbiol 72: 7554-7558. doi: 10.1128/AEM.01633-06.
5. Harmsen M, Lappann M, Knøchel S, Molin S. 2010. Role of extracellular DNA during biofilm formation by Listeria monocytogenes. Appl Environ Microbiol 76: 2271-2279. doi: 10.1128/AEM.02361-09.
6. Ishikawa E, Ishikawa T, Morita YS, Toyonaga K, Yamada H, Takeuchi O, Kinoshita T, Akira S, Yoshikai Y, Yamasaki S. 2009. Direct recognition of the mycobacterial glycolipid, trehalose dimycolate, by C-type lectin Mincle. J Exp Med 206: 2879-2888. doi: 10.1084/jem.20091750.

7. Osada-Oka M, Tateishi Y, Hirayama Y, Ozeki Y, Niki M, Kitada S, Maekura R, Tsujimura K, Koide Y, Ohara N, Yamamoto T, Kobayashi K, Matsumoto S. 2013. Antigen 85A and mycobacterial DNA-binding protein 1 are targets of immunoglobulin G in individuals with past tuberculosis. Microbiol Immunol 57:30–37. doi: 10.1111/j.1348-0421.2012.12005.x.
8. Menozzi FD, Bischoff R, Fort E, Brennan MJ, Locht C. 1998. Molecular characterization of the mycobacterial heparin-binding hemagglutinin, a mycobacterial adhesin. Proc Natl Acad Sci U S A 95:12625–12630. https://doi.org/10.1073/pnas.95.21.12625.
9. Katsube T, Matsumoto S, Taktsuka M, Okuyama M, Ozeki Y, Naito M, Nishiuchi Y, Fujiwara N, Yoshimura M, Tsuboi T, Torii M, Oshitani B, Arakawa T, Kobayashi K. 2007. Control of cell wall assembly by a histone-like protein in Mycobacteria. J Bacteriol 189: 8241−8249. doi: 10.1128/JB.00550-07.
10. Matsumoto S, Yukitake H, Furugen M, Matsuo T, Minta T, Yamada T. 1999. Identification of a novel DNA-binding protein from Mycobacteriuma bovis Bacillus Calmette-Guérin. Microbiol Immunol. 43:1027−1036. doi: 10.1111/j.1348-0421.1999.tb01232.x
11. Veale MF, Healey G, Sparrow RL. 2011. Effect of additive solutions on red blood cell (RBC) membrane properties of stored RBCs prepared from whole blood held for 24 hours at room temperature. Transfusion 51 Supplement 1: 25S-33S. doi: 10.1111/j.1537-2995.2010.02960.x.

Figure S1

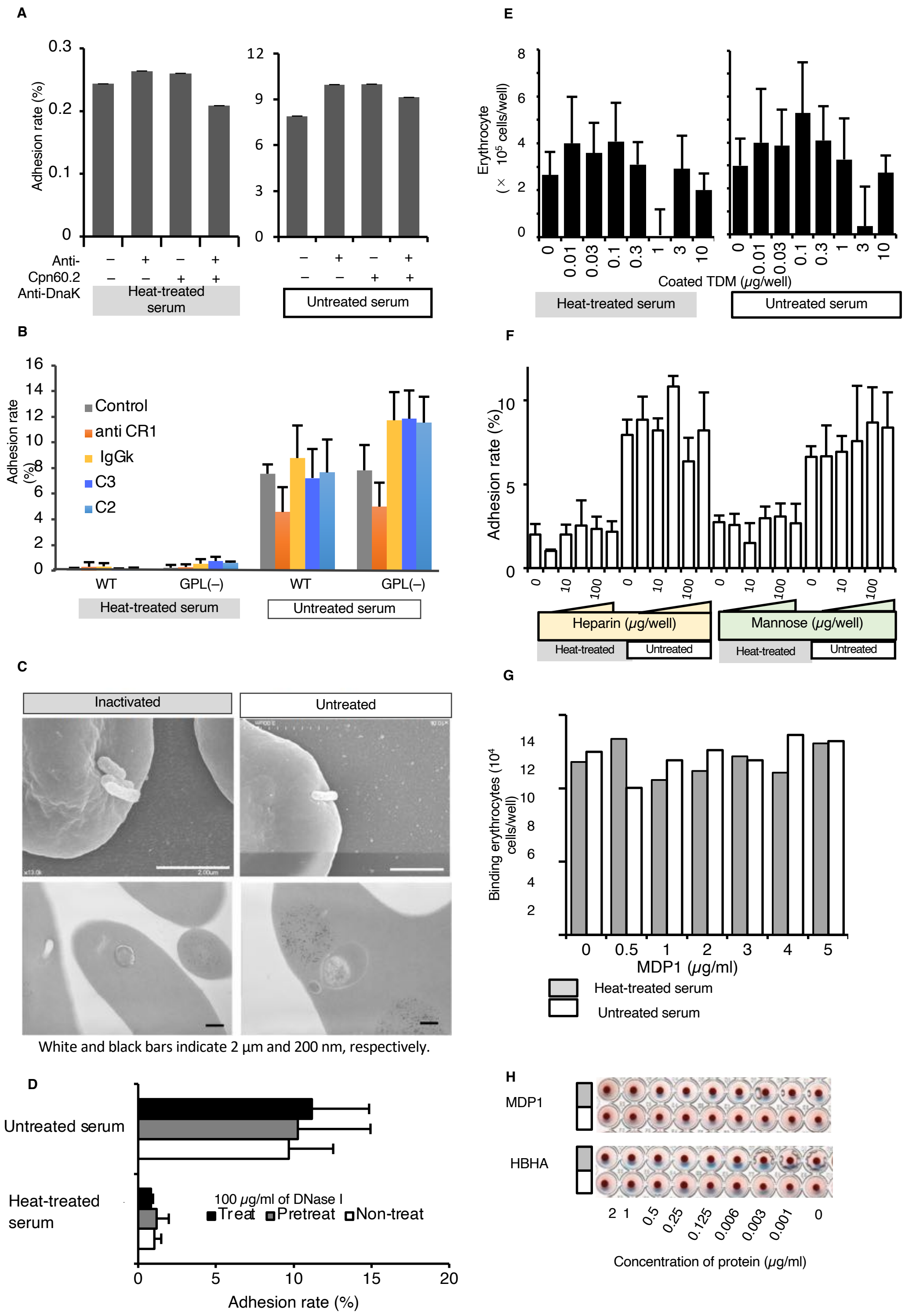

White and black bars indicate 2 μm and 200 nm, respectively.

Figure S1. Various candidate adhesin molecules of *Mycobacterium avium* subsp. *hominissuis* (MAH) did not affect the MAH attachment to human erythrocytes. (A) Pre-treatment with anti-DnaK and anti-Cpn60.2 antibodies did not reduce the adhesion rate of MAH to erythrocytes. (B, C) MAH104R, a GPL-deficient strain, also adhered to and invaded human erythrocytes according to the adhesion test (B) and SEM and TEM observations (C). Culture medium was supplemented with heat-treated (left) or untreated serum (right). (D) Extracellular DNA did not affect the adhesion rate. (E) Erythrocytes did not adhere to TDM-coated plates. (F) Heparin did not inhibit the attachment to human erythrocytes, nor did mannose. (G) Erythrocytes did not adhere to the MDP1-coated plates. The number of binding erythrocytes was counted by measuring the absorption of 405 nm after hemolysis. (H) Hemagglutination did not occur on MDP1/HBHA-coated plates. The plates were coated with indicated amounts of protein. The data shown are representative of multiple independent experiments. All error bars indicate SDs (n = 4).

Figure S2

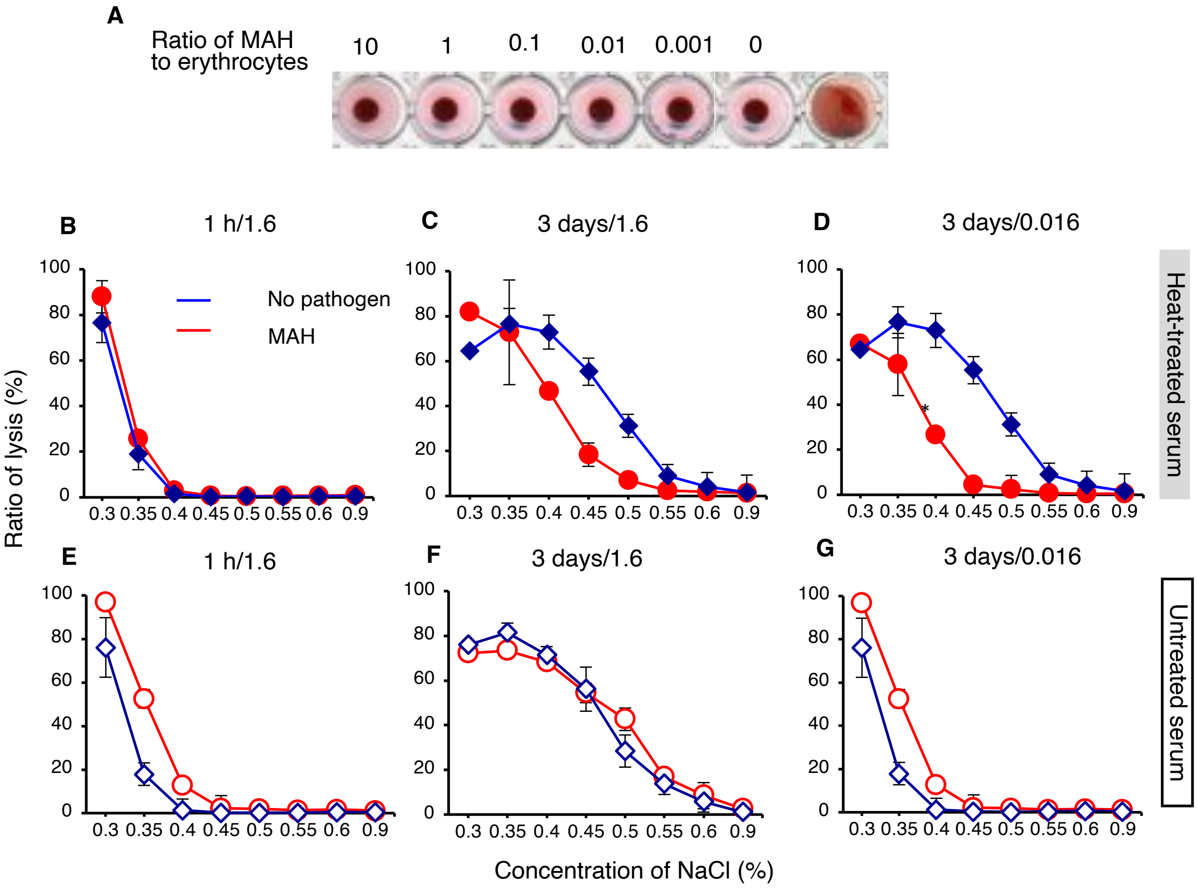

Figure S2. Erythrocytes with attached MAH did not cause hemagglutination or a significant reduction in the resistance to osmotic fragility. (A) Hemagglutination did not occur even after 5 h incubation at room temperature. Hemagglutination was observed in the case of a positive control performed by adding O-type serum into AB-type erythrocytes. (B–G) Resistance to osmotic fragility by erythrocytes. The inserted numbers indicate the incubation period or inoculation ratio of MAH cells to erythrocytes. **P* = 0.0333, as determined by one-way repeated measures ANOVA. All error bars indicate SDs (*n* = 4).

Figure S3

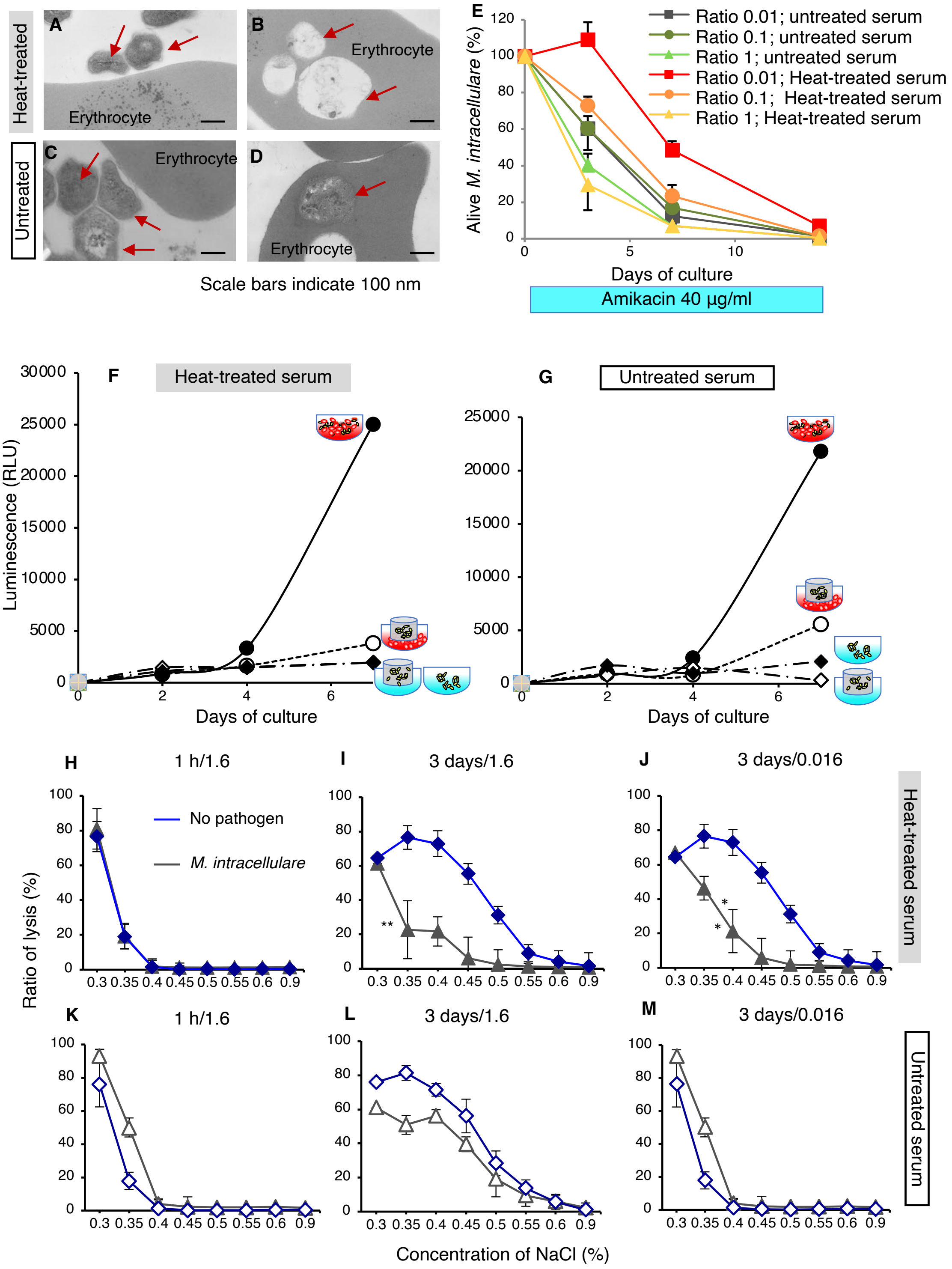

A
B
C
D
Heat-treated
Untreated
Erythrocyte
Scale bars indicate 100 nm
E
Alive M. intracellulare (%)
Ratio 0.01; untreated serum
Ratio 0.1; untreated serum
Ratio 1; untreated serum
Ratio 0.01; Heat-treated serum
Ratio 0.1; Heat-treated serum
Ratio 1; Heat-treated serum
Days of culture
Amikacin 40 µg/ml
F
Heat-treated serum
G
Untreated serum
Luminescence (RLU)
Days of culture
H
1 h/1.6
I
3 days/1.6
J
3 days/0.016
No pathogen
M. intracellulare
Ratio of lysis (%)
Heat-treated serum
K
1 h/1.6
L
3 days/1.6
M
3 days/0.016
Untreated serum
Concentration of NaCl (%)

Figure S3. Mycobacteria needed to directly attach to erythrocytes to multiply in a similar way to MAH. (A–D) BCG attached to (A, C) and invaded (B, D) erythrocytes, based on TEM observations. BCG organisms (arrows) were co-cultured with erythrocytes for 1 h. (E) Reduction of live *M. intracellulare* 198 cells during amikacin treatment. The *M. intracellulare*: erythrocyte inoculation ratios were 0.01, 0.1, and 1. The error bars indicate SDs (n = 4). (F, G) Promotion of extracellular *M. tuberculosis* growth via its direct attachment to erythrocytes. The culture conditions used are indicated in panels F and G. *M. tuberculosis* co-cultured with erythrocytes (closed circles); MAH separated from erythrocytes via a CI, (open circles); absence of erythrocytes with or without a CI (open and closed diamonds, respectively). (H–M) Resistance to osmotic fragility by erythrocytes. **P = 0.002 (I) and *P = 0.0182 (J), as determined by one-way repeated measures ANOVA. All error bars indicate SDs (n = 4).

Figure S4

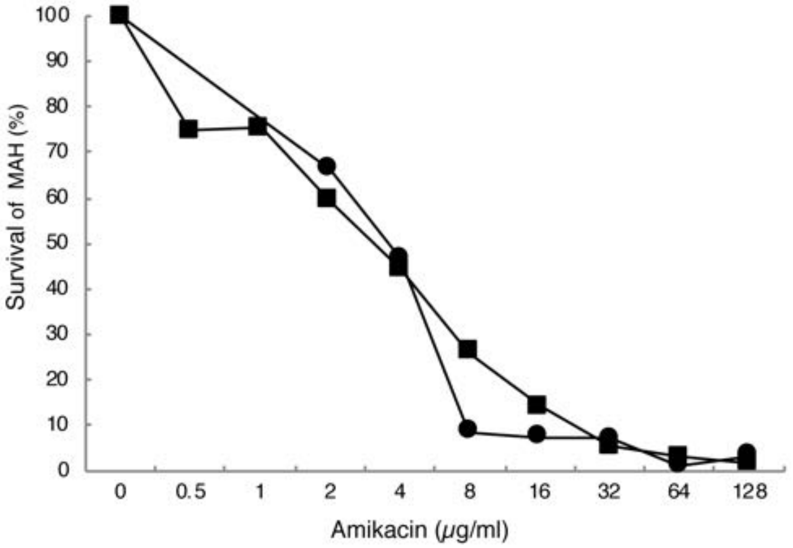

Figure S4. Amikacin possessed weak bactericidal activity against MAH. Amikacin decreased the number of viable MAH cells in the culture medium after a seven-day treatment. The culture medium comprised RPMI supplemented with 10% untreated serum (squares) or 10% heat-treated serum (circle).

Table S1

Table S1. MIC and MBC values of amikacin against MAH

Medium		MIC or MBC (μg/ml)		
		Middlebrook 7H9 broth	RPMI 1640 with heat-treated serum	RPMI 1640 with untreated serum
MAH 104	MIC	8	–*	–*
	MBC	>128	>128	>128

MIC values were determined on day 7.

*Cases where MIC values were not determined because MAH 104 did not grow even in the absence of amikacin in the RPMI 1640 medium
